# Reviewed article: Alves MN, Gigante DP, Mintem GC, Santos FS. Cross-sectional study of dietary practices from the Dietary Guidelines for the Brazilian Population among users of a Basic Health Unit in Pelotas, 2022. Epidemiol Serv Saude. 2025:34;e20240420

**DOI:** 10.1590/S2237-96222025v34e20240420.a

**Published:** 2025-08-04

**Authors:** Mariana Lopes

**Affiliations:** Universidade Federal da Paraíba, Nutrição

**Table:** 

Rating	Excellent	Good	Average	Below average	Poor
Interest				✓	
Quality					✓
Originality			✓		
Overall				✓	

**Table:** 

Questionnaire	Yes	No	Not applicable
Does the manuscript contain new and significant information to justify publication?	✓		
Does the Abstract (Summary) clearly and accurately describe the content of the article?		✓	
Is the problem significant and concisely stated?		✓	
Are the methods described comprehensively?		✓	
Are the interpretations and conclusions justified by the results?		✓	
Is adequate reference made to other work in the field?	✓		

## Manuscript Structure

Length of article is: Too little

Number of tables is: Adequate

Number of figures is: Adequate

Please state any conflict(s) of interest that you have in relation to the review of this paper (state “none” if this is not applicable): None

## Comments

1) GERAL:

- Alinhar em todo o texto incluindo no título a substituição do termo Atenção Básica para Atenção Primária à Saúde. Embora sinônimos, já se observa uma tendência do Ministério da Saúde quanto ao uso do termo Atenção Primária à Saúde;

2) RESUMO

• Excluir elementos de introdução do item objetivo. Começar com “Descrever”;

• Excluir elementos de métodos do objetivo no lugar das variáveis “m sexo, idade, raça/cor da pele, escolaridade, renda per capita, tabagismo e prática de atividade física” usar “analisar sua associação com fatores sociodemográficos e hábitos de vida”;

• Na seção método indicar em qual cidade e adicionar informações sobre análise de dados incluindo a forma de avaliação da referida escala;

• Na seção resultados adicionar números dos achados;

• Concluir com as implicações mais amplas dos achados do estudo para a saúde pública.

3) INTRODUÇÃO

• A introdução deve ser expandida para explicar a contribuição do estudo para o estado da arte sobre o assunto. Assim torna-se necessário melhor justificar o estudo da adesão as recomendações do Guia

4) MÉTODOS

• Talvez o item “Fontes de dados e mensuração” possa ser denominado “coleta de dados” e melhor descrever quais os dados coletados e quais as medidas de controle de qualidade dos dados empregados;

• Trata-se de um estudo com amostra de conveniência certo? Esclarecer no texto e indicar o porque foi escolhida a referida unidade;

• Pode ser interessante para os leitores que não conhecem a referida cidade e estado, que essa seja descrita em termos populacionais, IDHM, cobertura do SUS ect.

• Variáveis:

- Pode ser interessante separar em: desfecho; explicativas

- Foi feita a referência ao uso da ferramenta eGuia. A referida ferramenta data de 2018. Contudo há relato que dados coletados são de 2015 a 2019 (Página 3, linha 50). Como foi aplicado o eGuia entre 2015 a 2017 se a ferramenta ainda não existia? Tinham essas informações nos prontuários? Ou os anos de 2015-2018 foram para selecionar quem seria entrevistado?

- Sugiro uso de variáveis de estilo de vida ou hábitos de vida no lugar de comportamentais. Talvez seja o termo mais adequado tendo em vista o que de fato foi coletado: fumo e atividade física;

- Add referência para uso do p<0,20. Para além da escolha estatística outras variáveis importantes para a associação (segundo literatura) foram testadas e mantidas no modelo menos quando não atingiram p<0,20?. É importante o uso de modelo teórico para definir as variáveis.

- Há nesse estudo potenciais confundires? Porque não foram adicionados ao modelo como ajuste?

- O projeto foi submetido aos comitês de ética da Universidade e da Prefeitura?

Detalhar.

5) RESULTADOS

• A seção se beneficiaria se não repetisse integralmente os resultados da tabela. Maior dinamicidade da escrita poderá tornas esse item potente;

• Também poderia ser interessante a descrição das práticas segundo as variáveis sociodemográficas e de estilo de vida. Essa análise poderia apoiar a decisão pelas variáveis do modelo;

• Tabela 2 – porque não foram analisadas diferenças entre as frequências?

• Sugere-se substituir o termo bruto por não ajustado

6) DISCUSSÃO

• Primeiro parágrafo apresentar os principais achados. Responder ao objetivo

• É importante ainda concluir: Quais são as implicações mais amplas dos resultados do estudo? Como essas descobertas poderiam informar e transformar políticas? 

